# Heart failure—emerging roles for the mitochondrial pyruvate carrier

**DOI:** 10.1038/s41418-020-00729-0

**Published:** 2021-01-20

**Authors:** Mariana Fernandez-Caggiano, Philip Eaton

**Affiliations:** The William Harvey Research Institute, Barts and The London School of Medicine and Dentistry, Queen Mary University of London, Charterhouse Square, London, EC1M 6BQ UK

**Keywords:** Oxidoreductases, Cardiomyopathies

## Abstract

The mitochondrial pyruvate carrier (MPC) is the entry point for the glycolytic end-product pyruvate to the mitochondria. MPC activity, which is controlled by its abundance and post-translational regulation, determines whether pyruvate is oxidised in the mitochondria or metabolised in the cytosol. MPC serves as a crucial metabolic branch point that determines the fate of pyruvate in the cell, enabling metabolic adaptations during health, such as exercise, or as a result of disease. Decreased MPC expression in several cancers limits the mitochondrial oxidation of pyruvate and contributes to lactate accumulation in the cytosol, highlighting its role as a contributing, causal mediator of the Warburg effect. Pyruvate is handled similarly in the failing heart where a large proportion of it is reduced to lactate in the cytosol instead of being fully oxidised in the mitochondria. Several recent studies have found that the MPC abundance was also reduced in failing human and mouse hearts that were characterised by maladaptive hypertrophic growth, emulating the anabolic scenario observed in some cancer cells. In this review we discuss the evidence implicating the MPC as an important, perhaps causal, mediator of heart failure progression.

## Facts

- Heart failure is a leading cause of death with an unmet therapeutic need.
- MPC expression is decreased in heart failure—causing carbon to be rerouted, including to anabolic, growth pathways.
- Maintaining MPC expression protects against pressure-overload-induced hypertrophy and heart failure.
- A low carbohydrate, ketogenic diet protects against cardiac dysfunction caused by decreased MPC expression.

## Open questions

- What mechanisms mediate loss of the MPC during cardiac stress that culminates in heart failure?
- Is MPC activity post-translationally regulated during cardiac stress?
- What mechanisms, in addition to carbon shunting into growth pathways, contribute to heart failure progression resulting from decreased MPC expression?

## Introduction

The molecular identification of the mitochondrial pyruvate carrier (MPC) by two independent groups in 2012 [1, 2] led to studies describing its role in the function and regulation of healthy cells. Importantly, work showing that the MPC is a crucial determinant of disease progression by altering carbon utilisation by cells has and continues to emerge. This includes studies showing that MPC abundance is an important determinant in the pathogenesis of hypertrophy and heart failure following stress scenarios that increase work load or cause tissue injury, for example following ischemia. Here we discuss the evidence implicating decreased MPC abundance as an important, likely causal, mediator of heart failure progression. Defining the mechanisms that regulate the abundance and activity of this carrier may lead to therapeutic opportunities, as this could highlight strategies for maintaining MPC expression to protect against progression to heart failure.

## MPC structure and function

The MPC is a 150 kilodalton multimeric protein complex in mammals that comprises two subunits, named MPC1 (12 kilodalton) and MPC2 (14 kilodalton), with an additional third MPC3 subunit (16 kilodalton) in the yeast *Saccharomyces cerevisiae* [1, 2]. The MPC is integrated in the inner mitochondrial membrane, with each subunit predicted to have three transmembrane α-helices. Despite immunoprecipitation and bioluminescence resonance energy transfer-based biosensor studies demonstrating that MPC2 can form homodimeric complexes [3], MPC1–MPC2 heterodimers have mostly been reported to constitute the functional carrier [1–5]. However, this concept was challenged by Nagampalli et al., who reported that human MPC2 reconstituted into proteoliposomes forms functional homo-oligomers that allow efficient pyruvate transport [6].

**Fig. 1:**
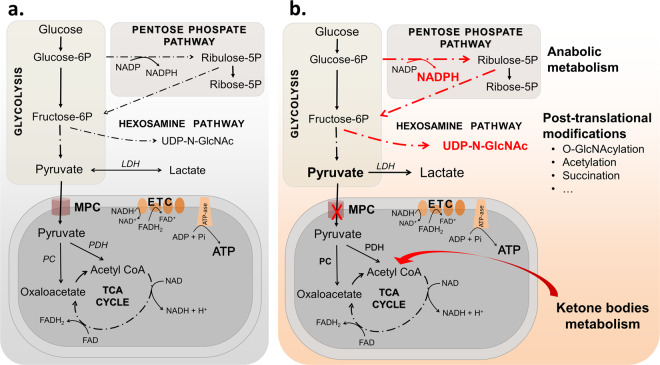
MPC as a metabolic branch point. **a** Glucose is converted to pyruvate via glycolysis. Pyruvate can be transported to the mitochondria through the MPC or reduced to lactate in the cytosol by lactate dehydrogenase (LDH). Inside the mitochondria, pyruvate can either be oxidised to acetyl-CoA by the pyruvate dehydrogenase (PDH) or carboxylated to oxaloacetate by pyruvate carboxylase (PC). Acetyl-CoA and oxaloacetate can enter the TCA cycle and generate the reducing equivalents NADH and FADH_2_ used by the electron transport chain (ETC) to generate ATP. **b** Decreased MPC expression alone is sufficient to cause metabolic cardiac remodelling, hypertrophic growth and ultimately heart failure. Mice genetically engineered to have decreased cardiomyocyte MPC1/2 divert glucose into the pentose phosphate pathway that generates five carbon sugars such as ribose 5-phosphate, a precursor for nucleotide synthesis and NADPH, which are necessary for anabolic metabolism and the growth of the myocardium. Shunting of fructose-6P from glycolysis towards the hexosamine biosynthetic pathway generates uridine diphosphate N-acetylglucosamine (UDP-N-GlcNAc) used by glycosyltransferases to transfer N-acetylglucosamine residues to proteins, termed O-GlcNAcylation. Other post-translational modifications are also likely to occur in cells as a result of changes in MPC abundance or activity due to the alterations in the TCA cycle or glycolysis caused by rerouting of pyruvate. ATP levels are maintained in heart with decreased MPC, perhaps because acetyl-CoA derived from ketone bodies is supplied to the TCA cycle, preserving the generation of reducing equivalents necessary for mitochondrial respiration.

Pyruvate obtained by glycolytic catabolism of glucose can be imported to the mitochondria through the MPC (Fig. 1a). Inside the mitochondria, pyruvate can either be oxidised to acetyl-CoA by the pyruvate dehydrogenase complex or carboxylated to oxaloacetate by pyruvate carboxylase. Both acetyl-CoA and oxaloacetate can enter the tricarboxylic acid cycle (TCA), generating reduced nicotinamide adenine dinucleotide (NADH) and flavin adenine dinucleotide (FADH) that are used by the electron transport chain to reduce molecular oxygen and generate the proton gradient necessary for adenosine triphosphate (ATP) synthesis. Alternatively, the oxaloacetate obtained by pyruvate carboxylation inside the mitochondria can be exported to the cytosol and contribute to the first step of gluconeogenesis, in which glucose is synthetised. Conversely, instead of being transported into the mitochondria, pyruvate can be reduced to lactate in the cytosol by lactate dehydrogenase (Fig. 1a). Although this reductive cytosolic reaction produces less ATP than that generated by mitochondrial oxidative phosphorylation, it serves as a mechanism that generates oxidised nicotinamide adenine dinucleotide (NAD^+^) required to maintain glycolysis at high rates. MPC abundance and activity is therefore a major determinant of the metabolic fate of pyruvate, providing an important regulatory mechanism that alters the biochemical products that are generated to impact on cellular homeostasis and as considered below, cell growth responses during stress.

## A potential role for MPC in the development of heart failure

The heart consumes a substantial amount of energy [7], consistent with its essential and continuous contractile pump function. Cardiac metabolism is tightly regulated and readily adapts under different developmental, nutritional, physiological or disease-related conditions. The healthy heart uses fatty acid oxidation to provide approximately 80% of its ATP, with the rest coming predominantly from glycolysis [8, 9]. This reliance on fatty acids for energy diminishes during heart failure [10], whilst myocardial glucose uptake and glycolysis is concomitantly augmented [11–13]. Despite this metabolic switch towards increased glucose uptake and glycolysis, the failing heart remains somewhat counterintuitively energy deficient. This is in part a result of pyruvate being handled differently by the failing heart. Instead of pyruvate being solely oxidised in the mitochondria a large proportion of it is instead reduced to lactate in the cytosol, which is inefficient in terms of ATP production and contributes to its deprived energy status [14, 15]. This metabolic scenario whereby cells increase glucose uptake and convert it to lactate via the reduction of pyruvate despite oxygen availability is reminiscent of the Warburg effect described extensively in many cancer cells [16]. In this context, decreased expression of the MPC increased cancer cell migration, promoted chemotherapy resistance and reduced patient survival [17–20], whereas overexpression of the carrier in cancer cells implanted into mice attenuated tumour growth [18]. Thus, decreased MPC expression in these studies limited the mitochondrial oxidation of pyruvate and contributed to lactate accumulation in the cytosol, highlighting its role as modulator of the Warburg effect.

As with cancer cells, the failing heart is also metabolically remodelled [21]. The healthy heart is metabolically flexible, allowing it to constantly energise the pumping of blood that is essential for host survival, even when environmental changes alter substrate or oxygen availability, or mandate increased cardiac output. As mentioned, the substrate preference of the heart switches towards glycolysis during scenarios such as ischemia and reperfusion or other stresses that culminate in pathological hypertrophy. Similarly, increased glycolysis and intermediates derived from glucose are also augmented in cancer cells, generating the biosynthetic substrates required for cell proliferation and growth. The similarities between metabolic remodelling in many cancers and the disease-induced hypertrophic heart that ultimately fails may indicate common maladaptive pathways mediate these pathologies. Below we discuss the potential consequences of variations in MPC expression and activity for the pathogenesis of pathological cardiac hypertrophy, as well as injury during myocardial ischemia and reperfusion.

## MPC in pathological cardiac hypertrophy

Downregulation of MPC1 and MPC2 was observed in failing human hearts and in mouse models of pathological cardiac hypertrophy [22, 23]. Similarly, decreasing MPC1/2 expression in adult mice using inducible transgenic methods was sufficient in itself to promote hypertrophy, providing evidence that the reduction in carrier abundance causatively mediates the pathological myocardial growth. This observation was supported by other studies where constitutive knockout of cardiomyocyte MPC1 or MPC2 in transgenic mice resulted in severe cardiac hypertrophy and heart failure [23, 24]. In line with observations from other cells or tissues [4, 18, 25], downregulation of one of the subunits in the cardiac-specific transgenic mice was associated with the concomitant loss of the other [22]. Interestingly, mice engineered to inducibly increase expression of MPC1 showed paralleled augmented MPC2 subunit expression [22]. These observations are consistent with the subunits reciprocally stabilising each other. Since the deletion of either subunit of MPC did not affect the transcription of the other, the regulation of the MPC complex may occur at the protein level [24].

Accumulation of some TCA cycle intermediates downstream of acetyl-CoA was observed in constitutive cardiac-specific MPC1 knockout or MPC2 knockout mice despite their decreased mitochondrial uptake of pyruvate [23, 24]. This may be because these mice with constitutive knockout of the carrier developed adaptive routes that supply carbon to the TCA cycle and so maintain the energy supply to the heart. Indeed, as discussed below, this adaptation might be mediated by ketone bodies or beta-oxidation of fatty acids replenishing acetyl-coA that sustains supply of substrates for oxidation in the TCA cycle to limit cardiac remodelling. Despite these metabolic adaptations, the knockout mice in both these studies developed pathological hypertrophy [24, 26], which was also observed in other transgenic mice with life-long cardiomyocyte knockout of MPC [22]. In contrast, transgenic mice in which cardiomyocyte MPC1 abundance was inducibly reduced only once they reached adulthood, which also caused hypertrophy, had decreased amounts of some TCA cycle intermediates in their hearts [22]. It remains unclear if the differences in TCA cycle profile between studies are because of the time in life when MPC abundance was decreased, or whether it is the magnitude of the reduced expression. It is notable that the experiments genetically inducing a reduction in MPC expression in mice after they reached adulthood were comparable to that observed in human failing hearts [22].

There are several potential mechanistic explanations for why decreased MPC expression leads to cardiac dysfunction. The simplest might be an inability of MPC knockout hearts to satisfy the energy demand of the myocardium due to the decreased transport and oxidation of pyruvate in the mitochondria. However, ATP and adenylate energy charge remained unchanged in hearts where MPC was deleted in adulthood [22, 24]. Thus, the metabolic remodelling that accompanies hearts with lower MPC1/2 levels does not impact ATP abundance. Thus, despite these metabolic changes decreasing carbon entry to the TCA cycle, the reducing equivalents generated appear sufficient to maintain mitochondrial respiration and consequent ATP generation. This observation is in agreement with previous studies that showed that perfusing hearts with different substrates did not alter their ATP levels [27]. As mentioned above, the failing heart is characterised by both a switch in fuel preference from fatty acids to glucose, as well as being energetic deficient. If the metabolic remodelling does not have a direct effect on cardiac energy provision, it might mediate other pathways which ultimately result in loss of cardiac efficiency that eventually compromises provision of ATP.

Alteration in MPC expression caused substantive changes in the metabolic fingerprint of the heart that may lead to post-translational protein modifications that mediate multiple cell signalling events (Fig. 1b) [28]. For example, variations in the TCA cycle intermediate fumarate, as may occur when MPC abundance is altered and anticipated to modulate protein succination that could mediate changes that accompany changes in carrier expression. Elevated O-GlcNAcylation of proteins was shown in MPC1 knockout hearts as a consequence of the shunt of glucose into the hexosamine biosynthetic pathway [24]. This is consistent with previous studies showing, increases in this post-translational modification as a posible cause of heart failure [29]. In addition to glucose being rerouted to the hexosamine pathway, it is also partly diverted to the pentose phosphate pathway as observed in mice where the MPC was deleted in adulthood. Rerouting of carbon to this anabolic pathway increased intermediates required for growth such as ribose-5-phosphate, a precursor for the synthesis of nucleotides and nucleic acids and erythrose-4-phosphate [22], which contributes to aromatic amino acid formation. Glucose shunting to the pentose phosphate pathway in mice with decreased MPC1/2 also increased NADPH, which is required for fatty acid and membrane synthesis necessary for myocardial growth (Fig. 1b).

A ketogenic diet can rescue the maladaptive cardiac remodelling that occurs when MPC expression is reduced [23, 24]. It is conceivable that this is because this diet impairs supply of glucose to cardiomyocytes from the liver, limiting glucose-6-phosphate formation and so growth involving the anabolic pentose phosphate pathway. Indeed, a high-fat, low-carbohydrate ketogenic diet that promoted weight loss and activity caused severe hepatic insulin resistance [30]. Similarly, chronic exposure of cardiomyocytes to the ketone body β-hydroxybutyrate induced their resistance to insulin, which as mentioned might limit carbon supply to the anabolic pentose phosphate pathway and so contribute to the associated protection against adverse cardiac remodelling [31]. Others have also observed attenuated pressure overload-induced heart failure by a ketogenic low-carbohydrate diet, but multiple alternate mechanisms were shown to contribute to this protection [31]. Nevertheless, such diets will replenish the TCA cycle by generation of acetyl-CoA via lipid or ketone metabolism without affecting mitochondrial respiration and ATP production. In fact, a ketogenic diet rescued embryonic lethality in germline MPC-deficient mice by providing acetyl-CoA directly to the TCA cycle, bypassing the need for a functional MPC [32]. Despite the efficacy of these diets in attenuating the development of cardiac hypertrophy caused by MPC deletion, a chronic ketogenic diet could lead to adverse health outcomes. For example, a ketogenic diet induced canonical signs of systemic stress and loss of well-being [33], and caused dyslipidemia, a pro-inflammatory state, hepatic steatosis, glucose intolerance and a reduction in beta and alpha cell mass [34]. This perhaps highlights the need for a more targeted approach. Inducibly maintaining MPC expression at healthy levels during transverse-aortic constriction in transgenic mice protected against cardiac hypertrophy [22], consistent with the rationale that drug interventions that increase expression or activity of the carrier, if they could be identified, may be therapeutic.

Although a reduction in MPC abundance was observed in mouse models of systolic heart failure [22–24], it was not in a Dahl salt-sensitive rat model of heart failure with preserved ejection fraction (HFpEF). In contrast, the myocardium of these rats exhibited a transient increase in expression of MPC1, but not MPC2, after 6 weeks of high salt [35]. Although this indicates a clear difference in the pathogenesis of these different types of heart failure, it is notable that ketone bodies, including when administered in the diet, protect against both these forms of cardiac dysfunction in mice [23, 24, 36]. Ketone bodies may be beneficial in human patients with pulmonary hypertension who exhibit HFpEF [37], with several planned clinical trials testing low-carbohydrate-induced ketogenesis or the ketone body 3-hydroybutyrate in patients with diastolic dysfunction planned or in progress. The benefits of such diets are complex as endogenous or exogenous ketones have multiple effects, especially the former that is achieved by reducing carbohydrate intake, and in some cases without calorie intake-matching control groups. Whilst this intricacy hampers our ability to definitively establish the molecular mechanisms of protection against heart failure, the fact that ketogenesis or dietary ketones were protective in cardiomyocyte-specific MPC knockout mice provides an indication that this is likely due to a direct impact on the metabolism and function of this contractile cell type that exhibits impaired diastolic relaxation in HFpEF. Thus, whilst ketones may exert multiple systemic effects that impact on myocardial structure and function, at least some of their therapeutic actions are likely via a direct impact on cardiomyocytes, correcting the metabolic and contractile dysfunction that arises as a result of decreased MPC expression during stress that culminates in hypertrophy and failure.

## MPC in ischemia reperfusion injury

MPC expression or activity in the heart might also alter cytosolic pH, an important modulator of cardiac excitation–contraction coupling, and a trigger of electrical arrhythmia. In healthy tissue, pyruvate derived from glycolysis is incorporated and metabolised in the mitochondria with the net production of protons from glucose metabolism being zero, as those produced by glycolysis are consumed by the TCA cycle [38]. However, if cytosolic pyruvate is not transported efficiently into the mitochondria, as may occur when MPC is less abundant or inhibited, it is reduced to lactate in the cytosol to cause acidosis [39]. During myocardial ischemia intracellular pH can decrease substantially, reaching ∼6.5 over time [40], with the associated extracellular acidosis being more marked [40, 41]. Intracellular acidosis leads to several potentially adverse events, including intracellular Na^+^ and Ca^2+^ overload [42], causing swelling due to osmosis and dysregulation of enzymes, including those of the myofilaments to potentially result in damaging hyper-contractile events. Protons also bind contractile proteins and decrease their sensitivity to Ca^2+^ to depress cardiac output, and may also initiate arrhythmias [43, 44]. The impact of MPC on myocardial injury during ischemia and reperfusion has been examined in isolated mouse hearts perfused with glucose, pyruvate and octanoate in the presence or absence of an inhibitor of the carrier, namely UK5099. This MPC inhibitor augmented infarction and reduced cardiac contractile output [45]. Interestingly, MPC was found at elevated levels in the surviving tissue within the peri-infarct border zone of pigs hearts 3 or 15 days after they were subjected to ischemia and reperfusion [45]. Thus, increased MPC expression may be a marker of surviving myocardium near the border of infarct zones. Furthermore, increased MPC abundance in the post-ischemic heart might represent an endogenous mechanism that enhances mitochondrial pyruvate uptake. Taking into account the metabolic switch to glycolysis after ischemia, an increased oxidation of pyruvate inside the mitochondria might be an important mechanism that maintains energy supply and myocardial tissue viability.

### What mechanisms control MPC expression and activity in the failing heart?

**Fig. 2:**
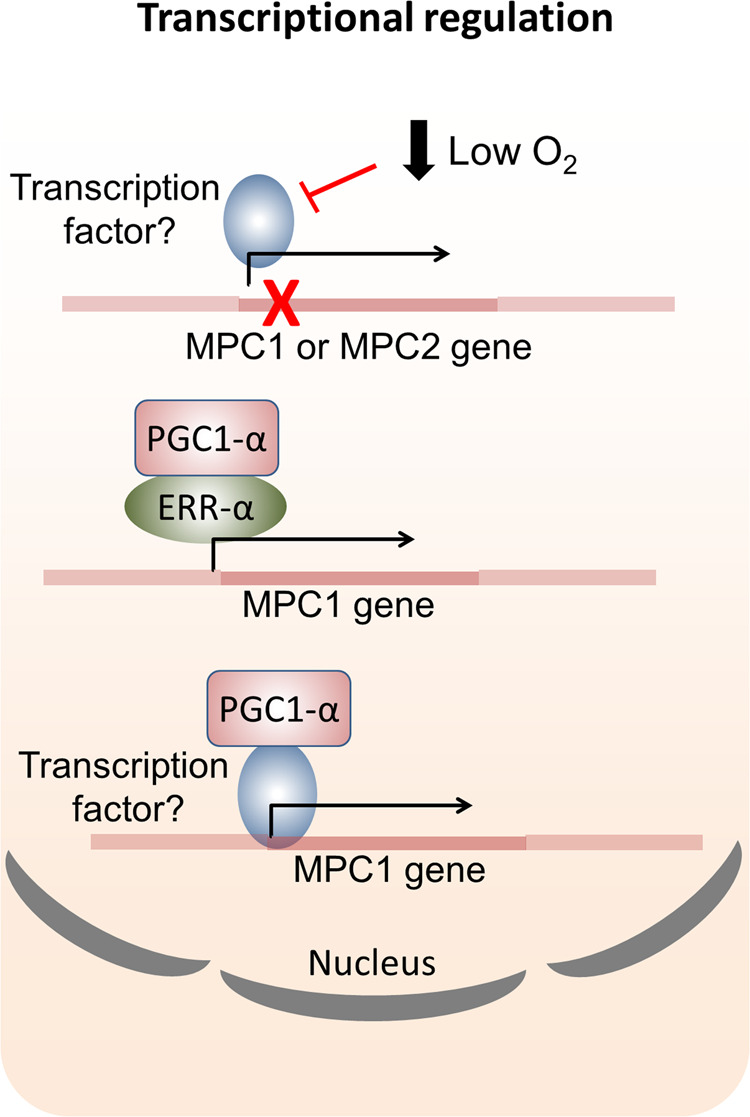
Transcriptional regulation of MPC. MPC gene expression is downregulated when the blood flow or oxygen is low. Oxygen or perhaps reactive species derived from it may directly or indirectly modulate transcription. The estrogen-related receptor alpha (ERR-α) is an established regulator of MPC1 transcription. ERR-α may be activated by the peroxisome proliferator-activated receptor γ coactivator-1α (PGC1-α), which has also been associated with MPC upregulation. It is possible that this coactivator additionally regulates yet unidentified transcription factors that modulate MPC1 gene expression.

As previously mentioned, downregulation of MPC was observed in failing, hypertrophic human hearts and in mouse models of pathological cardiac hypertrophy [22]. However, the mechanisms underlying MPC downregulation remain incompletely understood. Oxygen concentration may be an important determinant of MPC abundance. Bowman et al. showed that MPC expression was strongly downregulated in the heart during late gestation when the blood flow and so oxygen delivery is reduced [46]. Lower MPC expression is also correlated with poor prognosis in several types of cancers, such as colon and kidney, where tumour growth can promote hypoxia [18]. Hypoxia similarly induced lactate secretion and glycolytic flux by downregulating MPC levels in human umbilical vein endothelial cells [47]. Considering these scenarios are associated with low oxygen, it is rational to speculate that MPC expression could be potentially regulated by paralleled alterations in abundance of oxidant species that can serve as regulatory signals. These environmental fluctuations might control diverse redox mechanisms involved directly or indirectly in the activity of a transcription factor and thus MPC transcription and abundance. Inhibition of estrogen-related receptor alpha (ERR-α) reduced pyruvate entry into mitochondria by blocking the expression of MPC1 in breast cancer cell lines [48]. Consistent with this, Dan et al. showed that MPC1 was upregulated by the peroxisome proliferator-activated receptor γ coactivator-1α (PGC1-α), a potent activator of the ERR-α [49] (Fig. 2). We speculate that PGC1-α and ERR-α, which are enriched in the cardiac muscle [50, 51], may regulate cardiac MPC expression, but this requires further investigation.

**Fig. 3:**
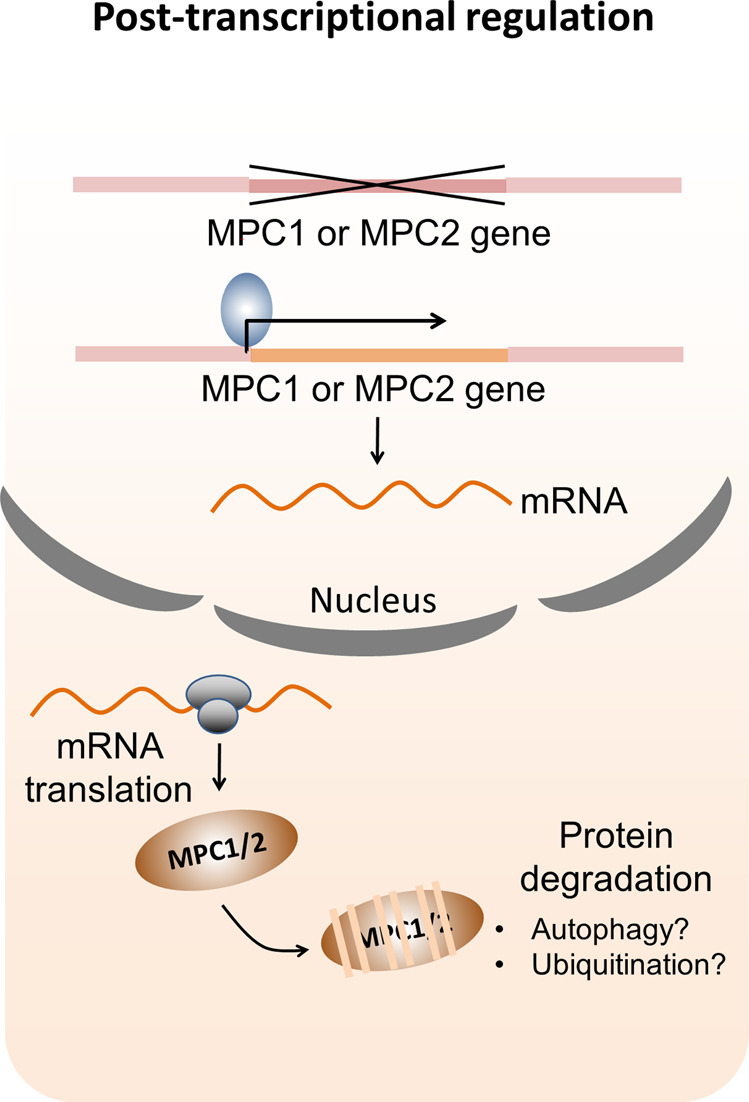
Post-transcriptional regulation of MPC. Deletion or low expression of either of the MPC subunits in cardiac tissue leads to downregulation of the other subunit without altering its transcript levels. This indicates that the turnover of MPC complex might be controlled by ubiquitination or autophagy processes happening at the protein level.

MPC activity and abundance may also be controlled by fluctuations in assembly and turnover of the complex. As mentioned above, decreased expression of either of the MPC subunits in cardiac tissue led to the downregulation of the other without altering its transcript levels [22]. This indicates that the reduction in MPC complex expression is likely regulated at the protein level (Fig. 3).

**Fig. 4:**
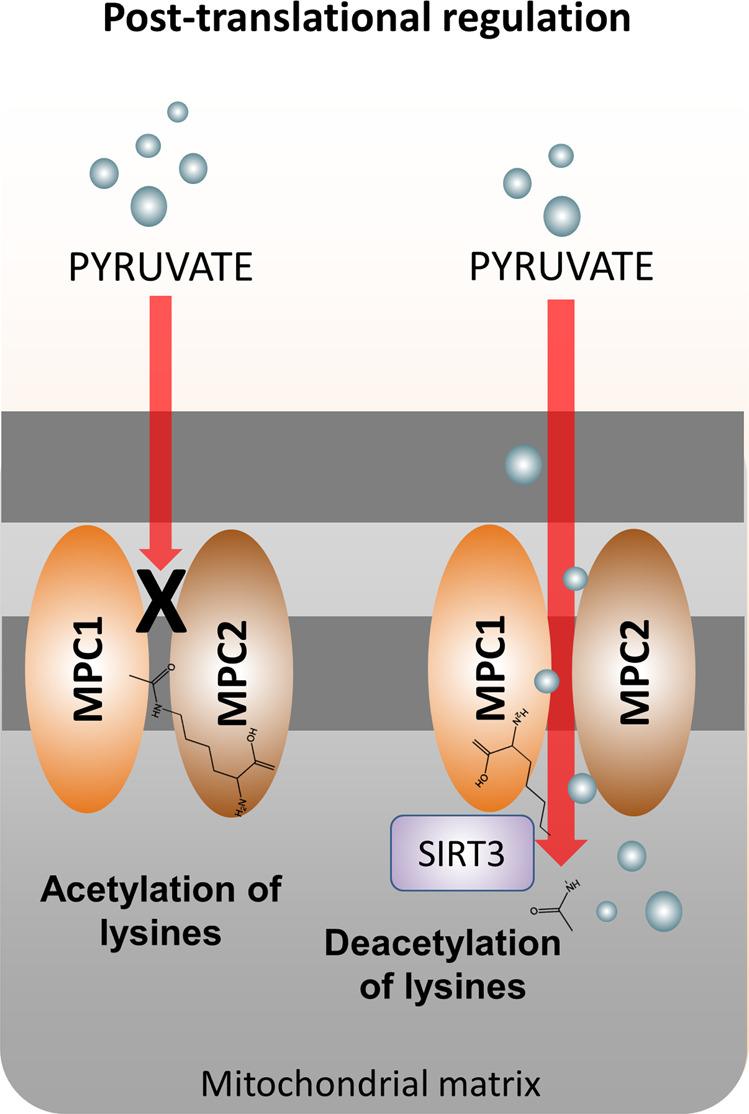
Post-translational regulation of MPC. Acetylation of lysines either in MPC1 or MPC2 reduced the rate of pyruvate transport to the mitochondria. Deacetylation of MPC1 by sirtuin 3 (SIRT3) can potentially increase the activity of MPC1 and pyruvate uptake to the mitochondria.

In addition to the transcriptional and post-transcriptional regulation, post-translational modifications in MPC can contribute to the modulation of pyruvate uptake. For example, acetylation of lysines 19 and 26 of MPC2 reduced the rate of pyruvate transport in the heart of Akita mice, a model of type 1 diabetes, by 70% [52]. In that study, Vadvalkar et al. showed the acetylating agent acetic anhydride inhibited pyruvate uptake in a similar manner to the MPC inhibitor α-cyano-4-hydroxycinnamate. Acetylation of the MPC1 subunit has not yet been described in the diabetic heart [52], despite being observed on lysines 45 and 46 in HEK293T or HCT15 cells [53]. Interestingly, sirtuin 3 (SIRT3) is a NAD^+^-dependent deacetylase able to bind and deacetylate MPC1 [53], potentially increasing the activity of the carrier. SIRT3 knockout mice exhibited increased glycolysis and decrease cardiac glucose oxidation [54], indicating pyruvate transport might be affected because of elevated MPC acetylation. Thus, SIRT3 might fine-tune MPC activity via deacetylation of lysines, mediating metabolic alterations in the heart (Fig. 4). In fact, it is widely reported that SIRT3 activation protects against cardiac hypertrophy, as well as ischemia and reperfusion injury by regulating the acetylation of mitochondrial proteins [55, 56]. Site directed mutation of lysines 19 and 26 in MPC2 to glutamine, which mimics acetylation, also decreased abundance of this subunit, indicating that this post-translational modification might also play a role in the stability of this subunit [52].

The hypoxia-inducible factor 1 (HIF-1) transcription factor, as with MPC, has a major impact on glucose handling. Although HIF-1 activation increases abundance of proteins that mediate glycolysis, it also induces pyruvate dehydrogenase kinase 1 expression to enhance the inhibitory phosphorylation of pyruvate dehydrogenase and repress mitochondrial utilisation of pyruvate in the TCA cycle [57]. MPC1 was increased in renal carcinoma cells during hypoxia and its abundance was negatively correlated with HIF-1 expression [58]. In contrast, human umbilical vein endothelial cells decreased their MPC1/2 abundance during hypoxia, with a concomitant decrease in the mRNA for each subunit indicating this is potentially because of decreased transcription [47]. Cardiomyocyte-specific MPC1 knockout mice had elevated 2-hydroxyglutarate [59], a growth-promoting onco-metabolite [60] that undergoes non-enzymatic conversion to 2-oxoglutarate to inhibit prolyl hydroxylase domain enzymes [61], which otherwise hydroxylates two conserved prolines in HIF-1 resulting in its ubiquitin-dependent proteosomal degradation. Indeed, cardiomyocyte-specific MPC1 knockout mice had increased myocardial amounts of the HIF-1 transcription targets such as lactate dehydrogenase A, pyruvate dehydrogenase kinase 1 and pyruvate kinase M2. Thus, the hypertrophy and failure observed in cardiomyocyte knockout mice MPC may be, at least in part, by 2-hydroxyglutarate activation of HIF-1 [59]. This and other mechanisms, such as the post-translational modifications discussed above, may contribute to the impaired metabolic flexibility of the diabetic heart discussed below.

### MPC and diabetic cardiomyopathy

HFpEF and systolic heart failure with reduced ejection fraction are increased in patients with diabetes mellitus and are not ameliorated by anti-hyperglycemic interventions [62]. Although streptozotocin-induced diabetic mice have increased myocardial β-hydroxybutyrate, which as discussed above is associated with protection from heart failure, the concomitant hyperglycaemia impairs ketone utilisation [63]. Acetylation of MPC2 lysines 19 and 26 in Akita diabetic mice inhibits cardiac pyruvate transport into myocardial mitochondria [52]. These mechanisms mediate the impaired metabolic flexibility of the diabetic heart and likely contribute to the pathogenesis of the associated cardiomyopathy. However, the precise causal mechanisms remain unclear, but are important to define because of the therapeutic opportunities this might offer. Whilst this review primarily focusses on cardiomyocyte MPC abundance and the etiology of heart failure, the contribution to MPC in other cell types and tissues should be noted, especially as many of those may also indirectly impact on the progression of the syndrome. For example, pancreatic beta-cell MPC activity regulates their sensing of glucose and coupled secretion of insulin, with blockade of the carrier attenuating release of the hormone to cause glucose intolerance [64, 65]. Insulin has systemic effects on multiple tissues and organs that modulate the heart and its susceptibility to failure, but there is added complexity because the cells in those systems also express the pyruvate carrier. This includes skeletal muscle, in which MPC was identified as a ‘whole-body carbon flux control point’, with decreased carrier expression reducing adiposity and increasing leanness that therefore has therapeutic implications for diabetes [66], and so progression to heart failure. MPC is also expressed in renal tubules and increasing its abundance, which is otherwise decreased in humans with diabetic nephropathy, might ameliorate the associated nephropathy [30], which together with diabetes are risk factors for heart failure. Thus, even with the use of cell type-specific genetic modulation of MPC expression, there are substantive difficulties in untangling the complexities of how carrier abundance in the cardiomyocyte or other cells types modulates the development of heart failure. Nevertheless, we are mindful that our studies inducibly sustaining cardiomyocyte MPC expression in adult hearts using genetic methods during pressure-overload protected against failure [22]. Thus, regardless of the complexity, pharmacotherapies that increase MPC abundance or activity are rational to identify and test as novel therapies for heart failure.

### Conclusions and perspectives

Since the identification of the molecular entity that is the MPC in 2012, an increasing number of studies continue to describe its role as a crucial metabolic branch point that controls how pyruvate is handled and the biochemical products that are derived from it. MPC is the entrance for pyruvate to the mitochondria and carrier activity is controlled by its abundance and post-translational modification, determining whether this substrate is oxidised in the mitochondria or metabolised in the cytosol. This explains why MPC is so important for the maintenance of metabolic and energy homeostasis.

In recent years MPC have been widely studied in the cancer field where evidence shows a loss in its abundance contributes to the Warburg effect that enhances cellular growth and division. Although there are fewer studies on the heart, a major message of this review is that decreased MPC expression is a characteristic feature of maladaptive hypertrophic grow in the myocardium. At the molecular level this is mediated, at least in part, by less pyruvate carbon entering the mitochondria for oxidation, but instead being diverted to the anabolic pentose phosphate or hexosamine biosynthetic pathways. These metabolic adaptations triggered by downregulation of MPC might lead to redox and post-translational changes that remain under-explored in hypertrophic cardiomyopathy, but also in other disease scenarios leading to heart failure. Inhibition of MPC or a reduction in its expression might also exacerbate the detrimental effects of lactate accumulation during cardiac ischemia. In this scenario, lactate accumulation promotes intracellular acidosis, leading to Na^+^ and Ca^2+^ overload that mediates cardiac dysfunction by multiple well-established mechanisms.

MPC is a key modulator of metabolic flexibility, but there are still many unanswered questions relating to its regulation as well as its role in the pathogenies of heart failure. Interventions that increase MPC activity may prove therapeutic by maintaining efficient energy production by oxidative phosphorylation, whilst also decreasing conversion of pyruvate to anabolites essential for maladaptive growth events. In view of advances in and access to metabolomics, as well as molecular tools for studying pyruvate transport by MPC, it would be reasonable to assume these questions may be answered sooner rather than later.
